# Supplementary material for: Multivariate emulation of computer simulators: model selection and diagnostics with application to a humanitarian relief model
Source: J R Stat Soc Ser C Appl Stat. 2016 Mar 1;65(4):483–505. doi: 10.1111/rssc.12141 (PMC4991306; doi:10.1111/rssc.12141)
Supplement: Supplementary file 1 — ‘Supplementary material for “Multivariate emulation of computer simulators: model selection and diagnostics with application to a humanitarian relief model”’. [file RSSC-65-483-s001.pdf]

Supplementary Material for "Multivariate emulation of computer simulators: model selection and diagnostics with application to a humanitarian relief model"

Antony M. Overstall
School of Mathematics & Statistics
University of Glasgow
Glasgow, UK

David C. Woods
Southampton Statistical Sciences Research Institute
University of Southampton
Southampton, UK

This document gives some more detail on the distributional results presented in this article.

1 Distributions

1.1 Inverse-Wishart distribution

If the $k \times k$ positive-definite matrix Σ has the $\mathrm{IW}_k(S, \delta)$ distribution, then

$$\pi(\Sigma) \propto |S|^{(\delta+k-1)/2} |\Sigma|^{-k-\delta/2} \exp\left\{ -\frac{1}{2} \mathrm{tr}\left(\Sigma^{-1} S \right) \right\}.$$

1.2 Matrix normal distribution

If the $n \times k$ matrix Y has the $\mathrm{MN}_{n,k}\left(M, \Sigma, \Omega\right)$ distribution, then

$$\pi(Y) \propto |\Sigma|^{-n/2} |\Omega|^{-k/2} \exp\left\{ -\frac{1}{2} \mathrm{tr}\left(\Sigma^{-1} \left(Y - M\right)^{\mathrm{T}} \Omega^{-1} \left(Y - M\right) \right) \right\}.$$

1.3 Matrix t distribution

If the $n \times k$ matrix Y has the $\mathrm{MT}_{n,k}(M, S, R, \delta)$ distribution, then

$$\pi(Y) \propto |S + (Y - M)^{\mathrm{T}} R^{-1} (Y - M)|^{-(n+k+\delta-1)/2} .$$

1.4 Matrix normal inverse-Wishart distribution

If the $m \times k$ and $k \times k$ matrices B and Σ have a $\mathrm{MNIW}_{m,k}(M, \Omega, S, \delta)$ joint distribution then

$$\pi(B, \Sigma) \propto |\Sigma|^{-k-(\delta+m)/2} \exp\left\{-\frac{1}{2}\mathrm{tr}\left(\Sigma^{-1}\left\{S + (B - M)^{\mathrm{T}} \Omega^{-1} (B - M)\right\}\right)\right\} .$$

2 Posterior distribution of B and Σ, given $\mathbf{r}$

Using Bayes' theorem, the joint posterior distribution of B and Σ (conditional on $\mathbf{r}$) is given by

$$\begin{aligned} & \pi(B, \Sigma|Y, \mathbf{r}) \\ \propto\ & \pi(Y|B, \Sigma, \mathbf{r})\pi(B, \Sigma|\mathbf{r}) \\ \propto\ & |\Sigma|^{-k-(\hat{\delta}+m)/2} \exp\left\{-\frac{1}{2}\mathrm{tr}\left(\Sigma^{-1}\left\{(Y - HB)^{\mathrm{T}} A^{-1} (Y - HB)\right.\right.\right. \\ & \left.\left.\left. +\ (B - M)^{\mathrm{T}} \Omega^{-1} (B - M) + S\right\}\right)\right\} . \end{aligned}$$

By expanding the two quadratic terms in the trace of the exponential and completing the square in terms involving B, we obtain

$$\pi(B, \Sigma|Y, \mathbf{r}) \propto |\Sigma|^{-k-(\hat{\delta}+m)/2} \exp\left\{-\frac{1}{2}\mathrm{tr}\left(\Sigma^{-1}\left\{\left(B - \hat{M}\right)^{\mathrm{T}} \hat{\Omega}^{-1}\left(B - \hat{M}\right) + \hat{S}\right\}\right)\right\} ,$$

and hence the joint posterior distribution of B and Σ (conditional on $\mathbf{r}$) is $\mathrm{MNIW}_{m,k}\left(\hat{M}, \hat{\Omega}, \hat{S}, \hat{\delta}\right)$. Hence

$$B|Y, \Sigma, \mathbf{r} \sim \mathrm{MN}_{m,k}\left(\hat{M}, \Sigma, \hat{\Omega}\right) , \tag{1}$$

and

$$\Sigma|Y, \mathbf{r} \sim \mathrm{IW}_k\left(\hat{S}, \hat{\delta}\right) . \tag{2}$$

This result is given for the case $A = I_n$ by Rougier (2007) and by Conti and O'Hagan (2010) for the case of weak prior information.

3 Conditional distribution of Y_0 given Y, B, Σ and $\mathbf{r}$

In equation (6) in the main manuscript, we state that the conditional distribution of Y_0 given Y (and B, Σ and $\mathbf{r}$) is matrix normal, given by equation (5) in the main manuscript. This follows from the definition of a conditional distribution, i.e.

$$\pi(Y_0|Y, B, \Sigma, \mathbf{r}) = \frac{\pi(Y, Y_0|B, \Sigma, \mathbf{r})}{\pi(Y|B, \Sigma, \mathbf{r})},$$

where the denominator is given by equation (2) from the main manuscript. Using the fact that

$$\begin{pmatrix} A & T \\ T^{\mathrm{T}} & A_0 \end{pmatrix}^{-1} = \begin{pmatrix} A^{-1} + A^{-1}TW^{-1}T^{\mathrm{T}}A^{-1} & -A^{-1}TW^{-1} \\ -W^{-1}T^{\mathrm{T}}A^{-1} & W^{-1} \end{pmatrix},$$

where $W = A_0 - T^{\mathrm{T}}A^{-1}T$, it follows from some matrix algebra that

$$Y_0|Y, B, \Sigma, \mathbf{r} \sim \mathrm{MN}_{n_0,k}\left(H_0B + T^{\mathrm{T}}A^{-1}(Y - HB), \Sigma, W\right).$$

4 Posterior predictive distribution of Y_0 given $\mathbf{r}$

The distribution of Y_0 given Y and $\mathbf{r}$ is found by integrating equation (6) in the main manuscript with respect to the joint posterior distribution of B and Σ. We first integrate with respect to B, whose posterior distribution, given Σ and $\mathbf{r}$, is matrix normal distribution (1). Since the distribution of Y_0 given Y, B, Σ and $\mathbf{r}$ is also matrix normal, then the distribution of Y_0 given Y, Σ and $\mathbf{r}$ will be matrix normal. It is easy to show that the column covariance matrix is Σ. Now consider the expectation

$$\begin{aligned} \mathrm{E}(Y_0|Y, \Sigma, \mathbf{r}) &= \mathrm{E}(\mathrm{E}(Y_0|Y, B, \Sigma, \mathbf{r})|Y, \Sigma, \mathbf{r}) \\ &= \mathrm{E}\left(H_0B + T^{\mathrm{T}}A^{-1}(Y - HB)|Y, \Sigma, \mathbf{r}\right) \\ &= H_0\hat{M} + T^{\mathrm{T}}A^{-1}\left(Y - H\hat{M}\right). \\ &= Q. \end{aligned}$$

Now consider the variance matrix of $\mathrm{vec}(Y_0)$:

$$
\begin{aligned}
\mathrm{var}\left(\mathrm{vec}\left(Y_0\right)|Y,\Sigma,\mathbf{r}\right) &= \mathrm{E}\left(\mathrm{var}\left(\mathrm{vec}\left(Y_0\right)|Y,B,\Sigma,\mathbf{r}\right)|Y,\Sigma,\mathbf{r}\right) \\
&\qquad + \mathrm{var}\left(\mathrm{E}\left(\mathrm{vec}\left(Y_0\right)|Y,B,\Sigma,\mathbf{r}\right)|Y,\Sigma,\mathbf{r}\right) \\
&= \mathrm{E}\left(\Sigma\otimes W|Y,\Sigma,\mathbf{r}\right) + \mathrm{var}\left(\mathrm{vec}\left(H_0B + T^{\mathrm{T}}A^{-1}\left(Y-HB\right)\right)|Y,\Sigma,\mathbf{r}\right) \\
&= \Sigma\otimes W + \mathrm{var}\left(\mathrm{vec}\left(\left(H_0 - T^TA^{-1}H\right)B\right)|Y,\Sigma,\mathbf{r}\right) \\
&= \Sigma\otimes W \\
&\quad + \left(I_k\otimes\left(H_0 - T^TA^{-1}H\right)\right)\mathrm{var}\left(\mathrm{vec}\left(B\right)|Y,\Sigma,\mathbf{r}\right)\left(I_k\otimes\left(H_0-T^TA^{-1}H\right)\right)^T \\
&= \Sigma\otimes\left(A_0 - T^{\mathrm{T}}A^{-1}T\right) \\
&\quad +\Sigma\otimes\left(\left(H_0 - T^{\mathrm{T}}A^{-1}H\right)\hat{\Omega}\left(H_0 - T^{\mathrm{T}}A^{-1}H\right)^{\mathrm{T}}\right) \\
&= \Sigma\otimes R
\end{aligned}
$$

which follows by using properties of Kronecker products (see, for example, Gentle, 2007, pages 72-74). Therefore the row covariance matrix of $Y_0|Y,\Sigma,\mathbf{r}$ is given by R and

$$
Y_0|Y,\Sigma,\mathbf{r}\sim \mathrm{MN}_{n_0,k}\left(Q,\Sigma,R\right)\,.
$$

Finally the distribution of $Y_0|Y,\mathbf{r}$ is given by integration with respect to the posterior distribution (2):

$$
\begin{aligned}
\pi(Y_0|Y,\mathbf{r}) &\propto \int \pi(Y_0|Y,\Sigma,\mathbf{r})\pi(\Sigma|Y,\mathbf{r})\mathrm{d}\Sigma \\
&\propto \int |\Sigma|^{-k-(\hat{\delta}+n_0)/2}\exp\left\{-\frac{1}{2}\mathrm{tr}\left(\Sigma^{-1}\left\{\hat{S} + \left(Y_0-Q\right)^{\mathrm{T}}R^{-1}\left(Y_0-Q\right)\right\}\right)\right\}\mathrm{d}\Sigma \\
&\propto \left|\hat{S} + \left(Y_0-Q\right)^{\mathrm{T}}R^{-1}\left(Y_0-Q\right)\right|^{-(n_0+k+\hat{\delta}-1)/2},
\end{aligned}
$$

which is the pdf of the $\mathrm{MT}_{n_0,k}\left(Q,\hat{S},R,\hat{\delta}\right)$ distribution.

5 Posterior predictive distribution for the total number of casualties

Recall $g(\mathbf{x}) = \sum_{i=1}^{k} f_i(\mathbf{x})$. Assuming a multivariate Gaussian process prior for $f(\mathbf{x}) = \{f_1(\mathbf{x}),\ldots,f_k(\mathbf{x})\}^{\mathrm{T}}$, a posteriori we can write $g(\mathbf{x}) = q(\mathbf{x}) + s(\mathbf{x})e$, where

$$
\begin{aligned}
q(\mathbf{x}) &= h(\mathbf{x})^{\mathrm{T}}\hat{M}\mathbf{1}_k + t(\mathbf{x})^{\mathrm{T}}A^{-1}(Y - H\hat{M})\mathbf{1}_k\,, \\
s(\mathbf{x}) &= \sqrt{\frac{\mathbf{1}_k^{\mathrm{T}}\hat{S}\mathbf{1}_k}{\hat{\delta}}r(\mathbf{x})}\,, \\
r(\mathbf{x}) &= c(\mathbf{x},\mathbf{x};\mathbf{r}) - t(\mathbf{x})^{\mathrm{T}}A^{-1}t(\mathbf{x}) + \left(h(\mathbf{x})^{\mathrm{T}} - t(\mathbf{x})^{\mathrm{T}}A^{-1}H\right)\hat{\Omega}\left(h(\mathbf{x})^{\mathrm{T}} - t(\mathbf{x})^{\mathrm{T}}A^{-1}H\right)^{\mathrm{T}}\,,
\end{aligned}
$$

e has a standard t-distribution with $\hat{\delta}$ degrees of freedom, and $t(\mathbf{x})$ is an $n \times 1$ vector with ith element given by $c(\mathbf{x}_i, \mathbf{x}; \mathbf{r})$.

6 Posterior predictive expectations for the total and partial variances

For a multivariate GP emulator, the posterior expected partial variances can be shown to have the form:

$$
\begin{aligned}
\mathrm{E}^*(V) &= \mathrm{E}\left[q(\mathbf{x})^2\right] - \mathrm{E}\left[q(\mathbf{x})\right]^2 + \frac{\hat{\delta}}{\hat{\delta}-2}\mathrm{E}\left[s(\mathbf{x})^2\right] , \\
\mathrm{E}^*(V_i) &= \mathrm{E}\left(\{\mathrm{E}\left[q(\mathbf{x})|x_i\right] - \mathrm{E}\left[q(\mathbf{x})\right]\}^2\right) + \frac{\hat{\delta}}{\hat{\delta}-2}\mathrm{E}\left(\{\mathrm{E}\left[s(\mathbf{x})|x_i\right] - \mathrm{E}\left[s(\mathbf{x})\right]\}^2\right) , \\
\mathrm{E}^*(V_{ij}) &= \mathrm{E}\left(\{\mathrm{E}\left[q(\mathbf{x})|x_i, x_j\right] - \mathrm{E}\left[q(\mathbf{x})|x_i\right] - \mathrm{E}\left[q(\mathbf{x})|x_j\right] + \mathrm{E}\left[q(\mathbf{x})\right]\}^2\right) \\
&\quad + \frac{\hat{\delta}}{\hat{\delta}-2}\mathrm{E}\left(\{\mathrm{E}\left[s(\mathbf{x})|x_i, x_j\right] - \mathrm{E}\left[s(\mathbf{x})|x_i\right] - \mathrm{E}\left[s(\mathbf{x})|x_j\right] + \mathrm{E}\left[s(\mathbf{x})\right]\}^2\right) .
\end{aligned}
$$

References

Conti, S. and O'Hagan, A. (2010) Bayesian emulation of complex multi-output and dynamic computer models. *Journal of Statistical Planning and Inference*, **140**, 640–651.

Gentle, J. E. (2007) *Matrix Algebra: Theory, Computations, and Applications in Statistics.* New York: Springer.

Rougier, J. C. (2007) Lightweight emulators for multivariate deterministic functions. *Tech. Rep. 07/02*, MUCM Technical Report, University of Durham.
